# Global burden of acute myocardial injury associated with COVID-19: A systematic review, meta-analysis, and meta-regression

**DOI:** 10.1016/j.amsu.2021.102594

**Published:** 2021-07-28

**Authors:** Semagn Mekonnen Abate, Bahiru Mantefardo, Solomon Nega, Yigrem Ali Chekole, Bivash Basu, Siraj Ahmed Ali, Moges Taddesse

**Affiliations:** a Department of Anesthesiology, College of Health Sciences and Medicine, Dilla University, Ethiopia; b Departemnt of Internal Medicine, College of Health Sciences and Medicine, Dilla University, Ethiopia; c Departemnt of Psychiatry, College of Health Sciences and Medicine, Dilla University, Ethiopia; d Public Health, College of Health Sciences and Medicine, Dilla University, Ethiopia

**Keywords:** Myocardial injury, Mortality, Prevalence

## Abstract

**Background:**

The body of evidence showed that there is a strong correlation between acute myocardial Injury and COVID-19 infection. However, the link between acute myocardial infection and COVID-19, the prevalence, reliability of diagnostic modalities, independent predictors, and clinical outcomes are still uncertain and a topic of debate. The current study was designed to determine the prevalence, determinants, and outcomes of acute myocardial injury based on a systematic review and meta-analysis the global published peer-reviewed works of literature.

**Methods:**

A comprehensive search was conducted in PubMed/Medline; Science direct, CINHAL, and LILACS from December 2019 to May 2021. All observational studies reporting the prevalence of AMI were included while case reports and reviews were excluded. The data were extracted with two independent authors in a customized format. The methodological quality of included studies was evaluated using the Newcastle-Ottawa appraisal tool.

**Results:**

A total of 397 articles were identified from different databases. Thirty-seven Articles with 21, 204 participants were included while seven studies were excluded. The meta-analysis revealed that the pooled prevalence of myocardial injury during the COVID-19 pandemic was 22.33 % (95 % CI: 17.86 to 26.81, 37).

**Conclusion:**

Our meta-analysis showed that mortality among patients with an acute myocardial injury during COVID-19 was more than four times more likely as compared to those without AMI. This necessitates a mitigating strategy to prevent and manage before its clinical outcomes getting worse.

## Highlights

∙ The meta-analysis revealed that more than one-fifth of patients with COVID-19 sustained AMI
∙The incidence of mortality among patients with AMI during COVID-19 pandemic was four times more likely as compared those without AMI
∙Prevalence of AMI was three times more likely in patients with history of Coronary artery disease
∙Smoking and hypertension were the independent predictors of acute myocardial Injury

Registration: This systematic review was registered in Prospero's international prospective register of systematic reviews (CRD42021257184).

## 1 Introduction

The severe acute respiratory syndrome virus-2 (SARS-CoV-2) virus that causes coronavirus disease 2019 (COVID-19) was identified in Wuhan, Hubei province of China in December 2019 by the Chinese Center for Disease and Prevention from the throat swab of a patient [1].

The coronavirus disease 2019 affects mainly the respiratory system through which the patient may end up with rapidly progressing pneumonia and acute respiratory distress syndrome [[1], [2], [3], [4], [5], [6], [7], [8], [9], [10], [11], [12]] despite current works of literature on the clinical manifestation of the gastrointestinal tract, cardiac, dermatologic, and central nervous system [[13], [14], [15], [16], [17], [18], [19], [20]]. However, the body of evidence showed that cardiovascular disorders particularly acute myocardial injury is getting attention as a result of associated short-term and long-term significant morbidity and mortality [6,[21], [22], [23], [24], [25], [26], [27], [28], [29], [30], [31], [32], [33], [34]].

The pathophysiological of myocardial injury associated with COVID-19 are still uncertain. However, there are many possible risk factors of myocardial injury in COVID-19 patients extensively described in the literature which includes but not limited to oxygen supply-demand imbalance due to respiratory failure, direct damage to the cardiomyocytes, systemic inflammation, myocardial interstitial fibrosis, interferon-mediated immune response, exaggerated cytokine response by Type 1 and 2 helper T cells [23,[35], [36], [37], [38]]. Besides, the risk of coronary thrombotic events from atherosclerotic plaque rupture has previously been shown to be increased during viral infections [[39], [40], [41]].

The diagnosis of myocardial injury in patients with COVID-19 with diagnostic imaging techniques was not described in published works of literature. However, cardiac biomarkers including highly sensitive Cardiac troponin I (hs-troponin I), creatinine kinase–myocardial band (CK-MB), myoglobin, and NT-natriuretic peptide were extensively described in the published literature [[21], [22], [23],[42], [43], [44], [45], [46], [47], [48]].

Different studies were conducted to investigate the predicting ability of cardiac biomarkers on in-hospital mortality [44,[49], [50], [51], [52], [53]]. But cardiac troponin showed consistent sensitivity and Specificity in different works of literature to predict in-hospital mortality [44,52,53]. A meta-analysis by Wibowo et al. conducted on the diagnostic performance of troponin showed that elevated troponin greater than 0.02 ng/ml was associated with a five-fold increase in mortality compared with patients without elevated troponin with sensitivity and septicity of 55 % and 80 % respectively [53]. A meta-analysis of 56 articles with 17 794 patients also revealed that patients with high troponin I more than 13.75 ng/L combined with either advanced age more than 60 years or elevated AST level more than 27.72 U/L was the most independent predictor of worse outcomes [52].

The incidence of myocardial injury associated with COVID-19 is very variable which ranges from 0.3 to 89 %. This variation is assumed to be linked to diagnostic biomarkers, presence of comorbidities, critical illness, and use of medications that are presumed to have high cardiac morbidity and mortality [30,31,34,40,46,50,[54], [55], [56], [57], [58]].

Studies showed that the prevalence of myocardial injury was strongly associated with the presence of different co-existing disease including cerebrovascular disease, coronary heart disease, hypertension, diabetes mellitus, obstructive pulmonary disease, chronic kidney disease, and using angiotensin-converting enzyme blocker and inhibitors [21,23,29,30,33,35,44,46,52,54,55,[57], [58], [59], [60], [61], [62]]. Besides, the mortality of patients associated with myocardial injury was correlated with pneumonia, acute respiratory distress syndrome, shock, heart failure, acute kidney injury, arrhythmia, coagulopathy, and advanced age. However, the incidence of myocardial injury, associated risk factors, short term, and long-term outcomes were variable in published literature and the evidence is not generalizable to the world [5,8,[10], [11], [12],^29^,^33^,^34^,^40^,^48^,^56^,[63], [64], [65], [66], [67], [68]]. In addition, most of the studies were conducted in China [5,8,[10], [11], [12],^33^,^34^,^48^,[66], [67], [68]]. Therefore, this systematic review, meta-analysis, and meta-regression were intended to provide pooled prevalence, determinants, and outcomes of Myocardial injury associated among COVID-19 patients by including recent studies conducted throughout the globe.

## 2 Methods

### 2.1 Protocol and registration

The systematic review and meta-analysis was conducted based on the Preferred Reporting Items for Systematic and meta-analysis (PRISMA) protocols [69], and the Meta-analysis Of Observational Studies in Epidemiology (MOOSE) checklist [70]. This systematic review and meta-analysis was registered in Prospero's international prospective register of systematic reviews (CRD42021257184) on May 27, 2021.

### 2.2 Eligibility criteria

All observational studies reporting the prevalence of myocardial injury among hospitalized patients with COVID-19 were included while studies that didn't report the prevalence of myocardial injury among hospitalized patients with COVID-19, articles that didn't report full information for data extraction, articles with different outcomes of interest, and Systemic review study design were excluded. The methodological quality of included studies was evaluated with ten points Newcastle-Ottawa appraisal tool as mentioned in the methodological quality assessment section and studies with a methodological score of less than fifty percent were also excluded. The primary outcomes of interest were the myocardial injury and mortality among hospitalized patients with COVID-19 worldwide. The prevalence of comorbidities, mean troponin value and lengths of hospital stay were secondary outcomes.

### 2.3 Search strategy

The search strategy was conducted to explore all available published and unpublished studies reporting myocardial injury among COVID-19 patients admitted to the hospital from December 2019 to May 2021 without language restrictions. A comprehensive search was employed in this review in different databases. An initial search on PubMed/Medline, Science Direct, CINHAL, and Cochrane Library was carried out followed by an analysis of the text words contained in Title/Abstract and indexed terms. A second search was undertaken by combining free text words and indexed terms with Boolean operators. The third search was conducted with the reference lists of all identified reports and articles for additional studies. Finally, an additional and grey literature search was conducted on Google scholars. The databases were searched with the following search terms using PICos (population, interest, context, and design) strategy by combining with AND, OR Boolean operators as COVID-19 OR novel coronavirus OR SARS-CoV-2 AND Myocardial injury OR myocardial damage OR myocardial infarction OR myocardial necrosis OR myocarditis OR myocardial dysfunction AND mortality OR death OR outcomes AND comorbidity OR complication AND prevalence OR incidence. The final search results were shown with the Prisma flow diagram (Fig. 1).

**Fig. 1:**
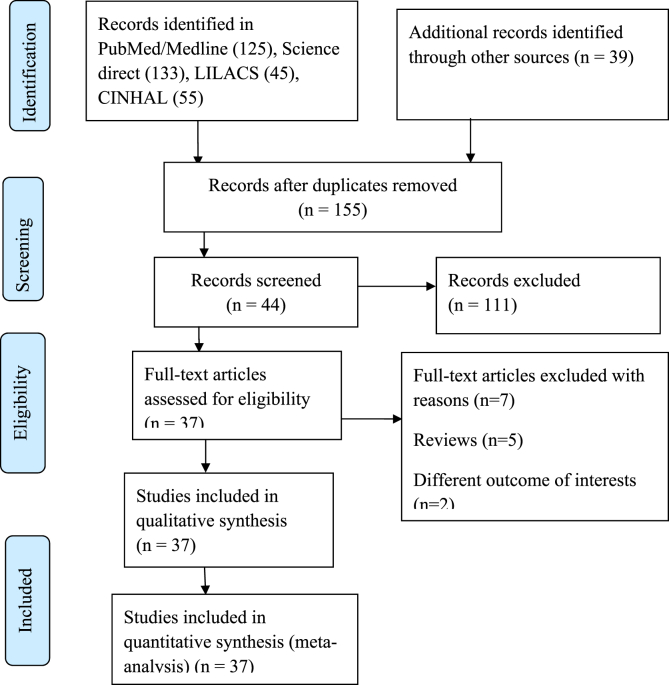
Prisma flow chart.

### 2.4 Data extraction

The data from each study were extracted by SA and YC independently with a customized format excel sheet. The disagreements between the two independent authors were resolved by the other authors. The extracted data included: Author names, country, date of publication, sample size, the prevalence of myocardial injury, mortality, the number of days in the hospital, presence of co-morbidities, mean and SD of cardiac biomarkers, and determinants. Finally, the data were then imported for analysis in R software version 4.0.2 and STATA 16.

### 2.5 Methodological quality assessment

Articles identified for retrieval were assessed by two independent Authors for methodological quality before inclusion in the review using Newcastle-Ottawa appraisal Scale (NOS) (Supplemental Table 1). The disagreements between the Authors appraising the articles were resolved through discussion. Articles with average scores greater than fifty percent were included for data extraction. Besides, the quality of this sytematic review and meta-analysis was evaluated with the Assessment of Multiple Systematic Reviews 2(AMSTAR) tool (figs1, figs2, figs3, figs4).

### 2.6 Data analysis

Data analysis was carried out in R statistical software version 4.0.2 and STATA 16. The pooled prevalence of myocardial injury, mortality, and length of hospital stay among hospitalized patients with COVID-19 was determined with a random effect model with restricted maximum likely hood (REML) as there was substantial heterogeneity. The Heterogeneity among the included studies was checked with forest plot, χ2 test, I^2^ test, and the p-values. Substantial heterogeneity among the included studies was investigated with subgroup analysis for categorical moderators (comorbidity, setting, country, and age group) and meta-regression for continuous covariates (mean troponin level, mean lengths of stay, mean age, and sample size) for outcomes extracted from more than ten studies. Publication bias was checked with a funnel plot and the objective diagnostic test was conducted with Egger's correlation, Begg's regression tests.

## 3 Results

### 3.1 Selection of studies

A total of 397 articles were identified from different databases with an initial search. Forty-four articles were selected for evaluation after the successive screening. Thirty-seven Articles with 21, 204 participants were included in the systematic review and Meta-Analysis while thirteen studies were excluded with reasons (Fig. 1).

### 3.2 Characteristics of included studies

Thirty-seven studies conducted on Coronavirus reporting prevalence and outcomes of acute myocardial injury with 21, 204 participants were included (Table 1). Seven studies were excluded with reasons.

**Table 1:** Description of included studies.

Author	Period	Country	AMI	Sample	age	quality
Cao et al., 2020 [7]	January 3 to February 1, 2020	China	15	102	72.5	8
Chen et al., 2020 [24]	January 13 to February 12, 2020	China	89	274	68.25	8
Deng et al., 2020 [44]	January 6 to February 20, 2020	China	42	112	67.5	8
Feng et al., 2020 [71]	January 1 to February 15, 2020	China	86	384	58.75	8
Ferrante et al., 2020 [64]	February 25 to April 2, 2020	Italy	123	332	74	10
Giustino et al., 2020 [46]	March 5 to May 2, 2020	Mulit-center	190	305	65.5	10
Gramegna et al., 2020 [47]	February 21 to April 1, 2020	Italy	7	26	66.25	7
Han et al., 2020 [72]	January 1 to February 18, 2020	China	34	273	58.12	7
Haung et al., 2020 [73]	Jan 2, 2020,	China	5	41	49.25	8
Lala et al., 2020 [54]	February 27th to April 12th, 2020	USA	985	2736	69.36	10
Li D et al., 2020 [36]	Jan 2020	China	39	182	72.75	8
Li et al., 2020 [26]	January 26 to February 5, 2020	China	119	548	64	10
Metkus et al., 2020 [29]	March 15 to June 11, 2020	USA	124	243	67.8	10
Modin et al., 2020 [56]	July 16, 2020	Denmark	17	5119	77	7
Popovic et al., 2020 [74]	February 26 to May 10th, 2020	France	11	83	62.6	7
Richardson et al., 2020 [61]	March 1 to April 4, 2020	China	801	5700	63.25	8
Shi et al., 2020 [33]	January 20 to February 10, 2020	China	82	416	69.25	10
Shi Q et al., 2020 [66]	January 1 to March 8, 2020	China	73	306	64	8
Shi S et al., 2020 [67]	January 1 to February 23, 2020	China	20	671	73.75	10
Stefanini et al., 2020 [40]	February 20 to March 30, 2020.	Italy	25	28	68	10
Tu et al., 2020 [75]	January 3 to February 24, 2020	China	18	174	71	6
Wang et al., 2020 [76]	January 1 to January 28, 2020	China	10	138	66.75	8
Wang Y et al., 2020 [9]	January 25 to February 25, 2020	China	111	344	57.5	7
Wei et al., 2020 [58]	January 16 to March 10, 2020	China	16	101	68.88	10
Wu et al., 2020 [10]	December 25, 2019 to January 26, 2020	China	9	201	51.25	10
Xiong et al., 2020 [12]	January 1 to March 10, 2020	China	85	131	64.3	8
Yang et al., 2020 [12]	December 2019 to Jan 26, 2020.	China	12	52	64.6	8
Aggarwal et al., 2020 [63]	January 31, 2020.	China	33	191	17.27749	7
Saleh et al., 2020 [65]	March to April 2020	USA	3	42	7.142857	10
Hong et al., 2020 [77]	March to May 2020	Iran	115	386	29.79275	10
Javanian et al., 2020 [78]	29-Mar-20	South Korea	11	98	11.22449	8
Lombardi et al., 2020 [79]	Feb 25 to March 12, 2020	Iran	14	100	14	9
Du et al., 2020 [25]	March 1 to April 9, 2020	Italy	45	614	7.32899	10
Xu et al., 2020 [80]	August 2020	China	14	179	7.821229	10

The methodological quality of included studies was moderate to high quality as depicted with the Newcastle-Ottawa Scale Appraisal tool for observational studies (Supplemental Table 1).

Twenty-four of the included studies were conducted in China while three studies were conducted in the USA and four in Italy. One study was conducted at a multi-country level. The remaining studies were conducted in France, Denmark, and South Korea. The mean age (±SD) of the.

Participants varied from 49.25 ± 4.25 to 77 ± 9 years.

All of the included studies reported a rate of acute myocardial injury and fourteen of the included studies reported mortality. The majority of included studies reported the presence of comorbidities including but not limited to hypertension, diabetes mellitus, cardiovascular disease, acute kidney injury, stroke, thrombosis, and acute respiratory distress syndrome while sixteen of the included studies reported current or history of cigarette smoking.

Some of the included studies reported markers of myocardial injury (Troponin I, Troponin T, Highly sensitive troponin I (hs-TnI), Creatinine Kinase Myocardial Band(CK-MB), Myoglobin, Brain Natriuretic Peptide (BNP).

Besides, inflammatory markers (white blood cells, reactive *C*-protein, Interleukin, etc), coagulation profiles (prothrombin time, thromboplastin, D-dimer), liver injury markers (Alanine aminotransferase, aspartate aminotransferase, lactate dehydrogenase), kidney biomarkers (creatinine, blood Urea nitrogen, glomerular filtration rate), and electrolytes were reported.

## 4 Meta-analysis

The meta-analysis was conducted to investigate the global pooled prevalence of acute myocardial injury and incidence of mortality among patients with the COVID-19 pandemic.

### 4.1 Prevalence of acute myocardial injury

The meta-analysis revealed that the pooled prevalence of myocardial injury among COVID-19 patients was 22.33 % (95 % CI: 17.86 to 26.81, 37 studies, 21 204 participants) (Fig. 2).

**Fig. 2:**
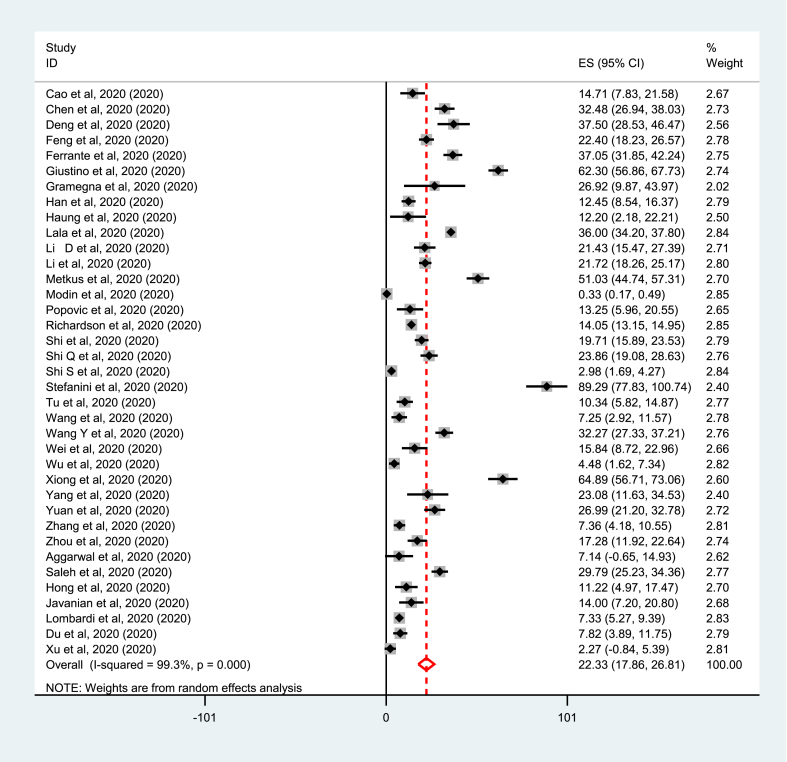
Forest plot for the prevalence of acute myocardial injury among patients with COVID-19: The midpoint of each line illustrates the prevalence; the horizontal line indicates the confidence interval, and the diamond shows the pooled prevalence.

### 4.2 Incidence of mortality

The incidence of mortality was extracted from each included study reporting mortality in patients with and without acute myocardial injury among COVID-19 patients. We did the meta-analysis with metan command in STATA and Meta package of R software with random effect and restricted maximum likelihood method. We reported the results of STATA as the pooled incidence is almost similar. The meta-analysis showed that the odds of mortality among patients with acute myocardial injury and COVID-19 was approximately nine times more likely as compared to those patients with COVID-19 without acute myocardial injury OR = 8.12 (95 % CI: 5.19 to 12.71, 14 studies) (Fig. 3).

**Fig. 3:**
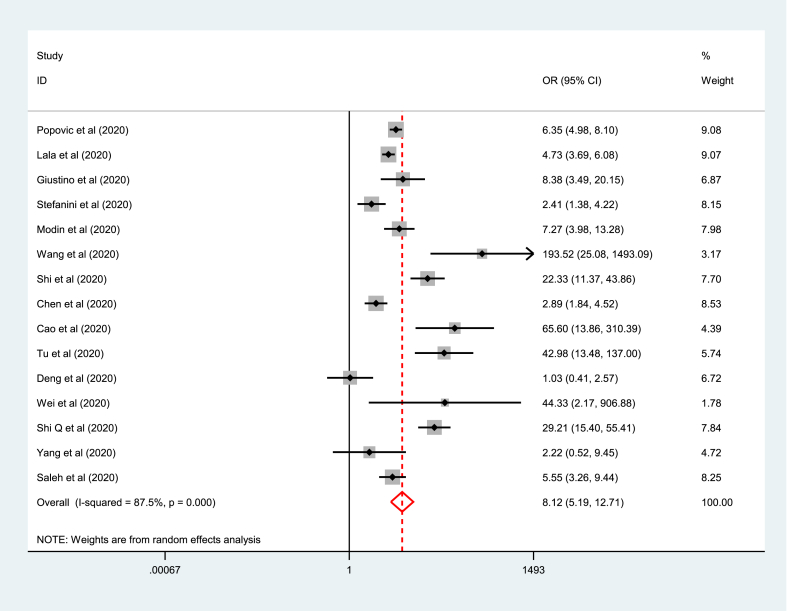
Forest plot for incidence of mortality among patients with an acute myocardial injury during COVID-19 pandemic: The midpoint of each line illustrates the prevalence; the horizontal line indicates the confidence interval, and the diamond shows the pooled incidence.

### 4.3 Sub-group analysis

The meta-analysis showed a substantial heterogeneity between the included studies as depicted with I-squared values and the corresponding p-value. As a result, sub-group analysis and meta-regression was conducted for continuous and categorical predictors. The subgroup analysis was conducted by the diagnostic cut point of highly sensitive Troponin I level for mortality. As it has been seen from the graph, the mortality among patients with acute myocardial injury didn't show a clinical difference with level of myocardial markers (Fig. 4).

**Fig. 4:**
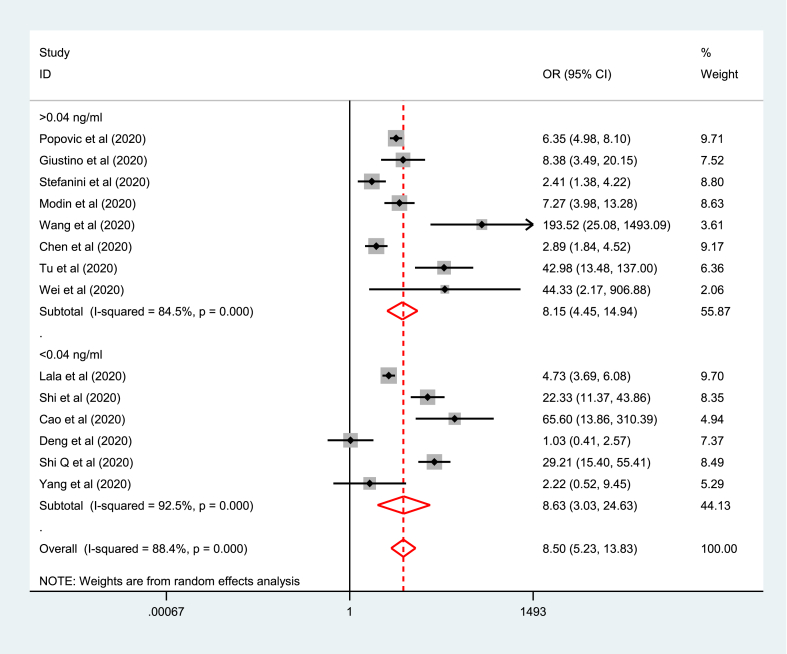
Forest plot for subgroup analysis of the incidence of acute myocardial injury by highly sensitive troponin I level among patients with COVID-19: The midpoint of each line illustrates the incidence; the horizontal line indicates the confidence interval, and the diamond shows the pooled incidence.

**Fig. 5:**
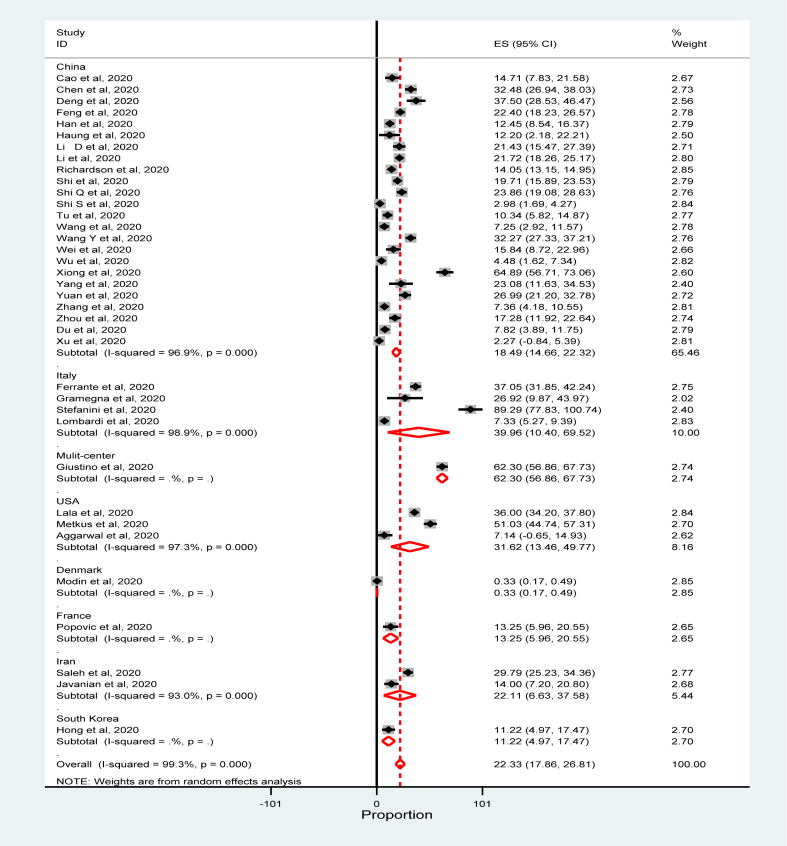
Forest plot for subgroup analysis of the prevalence of acute myocardial injury among patients with COVID-19: The midpoint of each line illustrates the prevalence; the horizontal line indicates the confidence interval, and the diamond shows the pooled prevalence.

The subgroup analysis of acute myocardial injury among patients with COVID-19 by country showed that the prevalence of acute myocardial injury was the highest in multi-countries followed by Italy and the USA: 62.30 % (95 % CI: 56.86 to 67.73), 39.96 % (95 % CI: 10.40 to 69.52), and 31.62 % (95 % CI: 13.46 to 49.77) respectively (Fig. 5).

### 4.4 Meta-regression

**Fig. 6:**
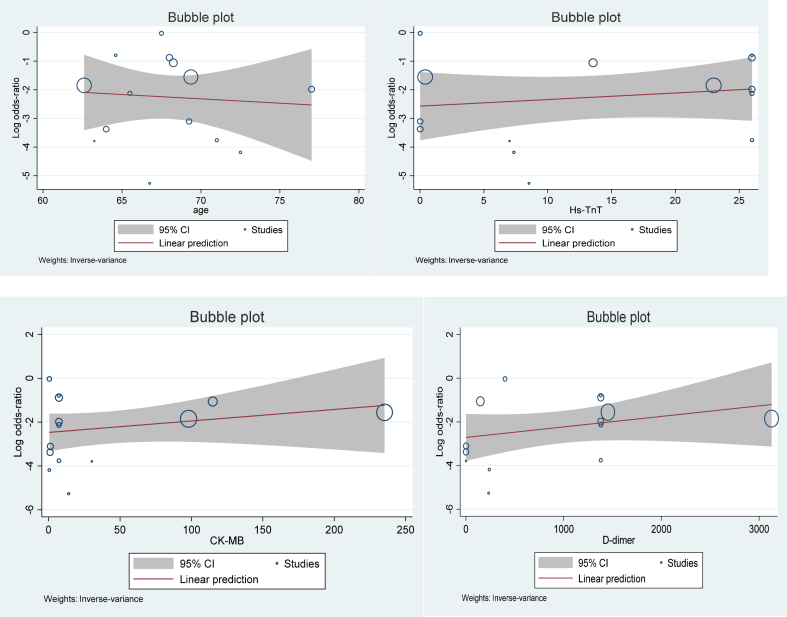
Meta-regression bubble plot for the continuous covariates (mean age, highly sensitive troponin I, Creatinine Kinase myocardial Band, D-dimer).

We conducted a meta-regression to investigate the sources of heterogeneity between the included studies with continuous covariates including mean age, the mean level of troponin I, troponin T, highly sensitive troponin I (hs-TnT I), creatinine kinase myocardial Band(CK-MB), Brain natriuretic peptide, and D-dimer. However, we failed to identify significant variability by each of the covariates on the effect size (Fig. 6).

### 4.5 Determinants mortality

**Fig. 7:**
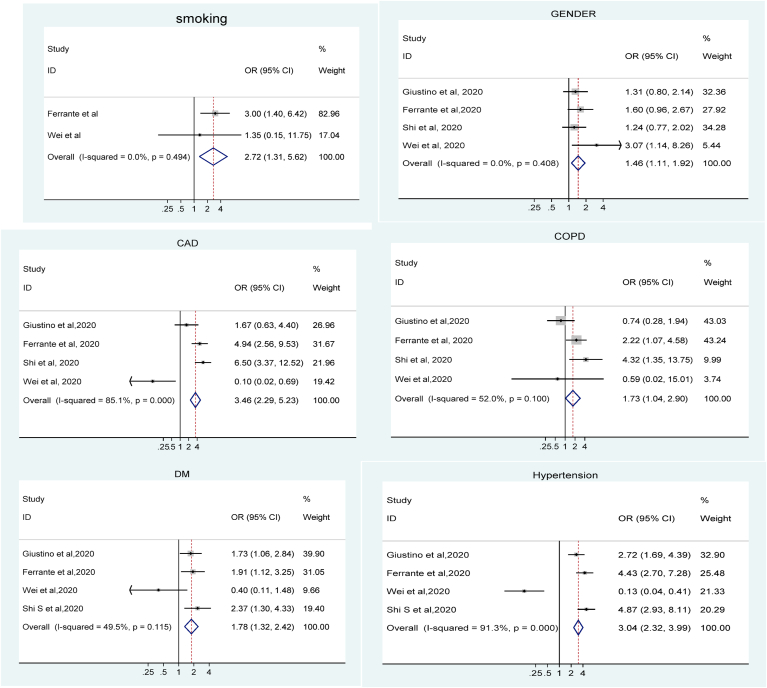
Forest plot for factor analysis for acute myocardial injury among patients with COVID-19: The midpoint of each line illustrates the prevalence; the horizontal line indicates the confidence interval, and the diamond shows the pooled odds ratio.

We conducted a factor analysis to investigate the independent predictor of mortality such as Coronary Artery Disease (CAD), Diabetes Mellitus (DM), hypertension, smoking, chronic obstructive pulmonary disease (COPD), and gender. This study revealed that the incidence of mortality was three times more likely in a patient with hypertension OR = 3.04 (95 % CI: 2.32 to 3.99). Besides, the incidence of mortality was three times more likely in a patient with a history of CAD OR = 3.48 (95 % CI: 2.29 to 5.23) (Fig. 7).

### 4.6 Sensitivity analysis and publication bias

**Fig. 8:**
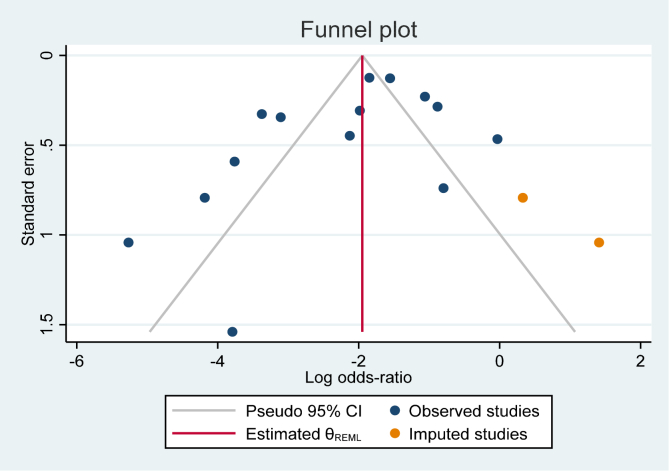
Funnel plot and trim fill to assess publication bias. The vertical line indicates the effect size whereas the diagonal line indicates the precision of individual studies with a 95 % confidence interval.

Sensitivity analysis was conducted to identify the most influential study on the pooled summary effect and we didn't find significant influencing on the summary effect. Besides, Publication biases was investigated with funnel plot asymmetry and egger's regression, Begg's rank correlation test, and trim fill method. The trim fill showed that two large standard error studies were missed but the rank correlation test didn't show a significant difference (*P*-value >0.05) (Fig. 8).

## 5 Discussion

Different works of the literature showed that there is a strong correlation between acute myocardial Injury and COVID-19 infection. New studies are being published from around the globe but the link between acute myocardial injury and COVID-19, the prevalence, reliability of diagnostic modalities, independent predictors, and clinical outcomes are still uncertain and a topic of debate [22,24,26,[32], [33], [34], [35],^41^,^44^,^50^,^53^,[57], [58], [59],^62^,^76^,[81], [82], [83], [84], [85]].

There is also a huge local and regional disparity in the incidence and mortality of patients with acute myocardial injury during the pandemic which might be related to the severity of disease, presence of comorbidities, age group of the participants, study period, and sample size of the studies [11,29,33,34,40,48,53,56,[63], [64], [65], [66], [67],^81^,[86], [87], [88], [89]].

This systematic review and meta-analysis was intended to investigate the global prevalence, mortality, and independent predictors of acute myocardial injury among patients with the COVID-19 pandemic. Our meta-analysis revealed that the pooled prevalence of acute myocardial injury in patients with COVID-19 was 22.33 (95 % CI: 17.86 to 26.81) which is relatively higher than other meta-analyses by Prasitlumkum et al. and Alzahrani et al., 20.0 % (95 % CI 16.1–23.8 %) and 20.62 respectively. This discrepancy might be related with inclusion of few studies and study setting where these authors included studies conducted mainly in china with Twenty-seven and nine studies respectively [81,83]. However, a meta-analysis by Zou et al. including sixteen studies where fourteen of them were from China showed higher incidence of acute myocardial infarction (24.4 %) among COVID-19 patients [90].

The incidence of mortality among patients with an acute myocardial injury during COVID-19 was eight times more likely as compared to patients without acute myocardial injury OR = 8.12 (95 % CI: 5.19 to 12.71, 14 studies). However, another meta-analyses by Fu et al. LI et al., and Zou et al. showed a higher incidence of mortality which is OR = 10.11 (95 % CI: 4.49 to 22.77), OR = 21.15 (95 % CI 10.19 to 43.94), and OR = 17.32 (95 % CI: 9.21 to 32.57) respectively [27,82,90] whereas a meta-analysis by Alzahrani et al. revealed a similar incidence of mortality to our meta-analysis [81]. Such a huge discrepancies in the incidence of mortality among patients with acute myocardial injury in COVID-19 patients among published meta-analysis might be related with inclusion of few studies, study setting, time of data collection, and inclusion of studies with sever case and comorbidities in some studies which can escalate the risk of myocardial injury and death.

This systematic review and meta-analysis identified the independent predictors of prevalence of acute myocardial injury among patients with COVID-19 infection which includes a history of smoking, being male gender, diabetes mellitus, hypertension, coronary artery disease, and chronic obstructive pulmonary disease. Patients with hypertension, coronary artery disease, and history of smoking were approximately three times more likely to develop acute myocardial injury among patients with COVID-19 OR = 3.04(95 % CI:2.32 to 3.99), 3.45(95 % CI: 2.29 to 5.23), and 2.72 (95 % CI: 1.31 to 5.62) respectively. While a met-analysis by Zou et al. revealed that the incidence of acute myocardial injury among patients with COVID-19 having hypertensive and Coronary artery disease was OR = 3.83 (95 % CI: 1.77 to 8.26 and COPD OR = 5.03 (95 % CI: 1.91–13.29) respectively [90]. Contrary to our meta-analysis, this study didn't show significance difference on the incidence of acute myocardial injury with coronary artery disease and diabetes mellitus [90].

Meta-regression was conducted to investigate the source of heterogeneity between the included studies and the variability on the effect size by mean age, sample size, and mean biomarkers. However, the independent factors didn't show any significant difference in the total heterogeneity between the included studies (P > 0.05).

### 5.1 Quality of evidence

The methodological quality of included studies was moderate to high quality as illustrated with the Newcastle-Ottawa scale appraisal tool for meta-analysis of observational studies. However, substantial heterogeneity associated with dissimilarities of included studies in the diagnosis of acute myocardial injury, study setting, age group, and sample size, could affect the allover quality of evidence.

### 5.2 Implication for practice

Body of evidence revealed that the prevalence of AMI and its outcomes was very high among hospitalized patients with COVID-19. Acute myocardial injury during COVID-19 is a huge challenge because cardiac biomarkers are unspecific while the diagnostic imaging including Echocardiography, angiography, and stress electrocardiogram may not be feasible particularly in severe and critically ill patients. Therefore, a mitigating strategy is required by different stakeholders to early diagnose and manage acute myocardial injury and its consequences.

### 5.3 The implication for further research

The meta-analysis revealed that the prevalence of acute myocardial injury and its outcomes was very high among hospitalized COVD-19. However, the included studies were too heterogeneous, low-powered, and cross-sectional studies also don't show a temporal relationship between the outcome and its determinants. Therefore, further observational and randomized controlled trials are required to provide a firm conclusion.

### 5.4 Limitation of the study

The meta-analysis included studies with moderate to high methodological quality. This meta-analysis overcomes some of the limitations of the previous meta-analysis which included small few studies which were conducted only in China. However, this meta-analysis included studies with were low-powered and the majority of included studies didn't report data on mortality, comorbidity, and risk factors to investigate the independent predictors. Besides, the included studies used different cut points for the diagnosis of acute myocardial injury with cardiac biomarkers and it would be difficult to provide conclusive evidence.

## 6 Conclusion

This meta-analysis showed that the prevalence of acute myocardial injury and mortality were very high among patients with COVID-19. Our meta-analysis showed that mortality among patients with an acute myocardial injury during COVID-19 was more than four times more likely as compared to those without AMI. Besides, patients with a history of smoking, acute coronary disease, chronic obstructive pulmonary disease, and hypertension were independent predictors of acute myocardial injury. This necessitates a mitigating strategy to prevent and manage before its clinical outcomes getting worse.

Declaration.

## Availability of data and materials

Data and material can be available where appropriate.

## Funding

No funding was obtained from any organization.

## Authors' contributions

SA and YC conceived the idea design of the project. SA, YC, BB, SN, and BM were involved in searching strategy, data extraction, quality assessment, analysis, and manuscript preparation. All authors read and approved the manuscript.

## Please state any conflicts of interest

The authors declared that there is no conflict of interest.

## Please state any sources of funding for your research

No funding was received.

## Ethical approval

Not applicable.

## Consent

Consent was not applicable as it was systematic review and meta-analysis.

## Author contribution

Semagn Mekonnen Abate conceived the idea and design of the project. Semagn Mekonnen Abate and Yigrem Ali Chekole, Bahiru Mantefardo, Solomon Nega, Bivash Basu, Siraj Ahmed and Moges Taddesse involved in data management, entry, analysis interpretation, and manuscript preparation. All authors read and approved the final manuscript.

## Registration of research studies

This review was registered in Prospero international prospective register of systematic reviews (CRD42021257184)).

## Guarantor

Semagn Mekonnen Abate, Corresponding Author, Assistant professor of Anesthesiology, Department of Anesthesiology, College of Health Sciences and Medicine Dilla University Tel:+251913864605Email:semmek17@gmail.com/semagnm@du.edu.et.

## Declaration of competing interest

The authors declare that there are no competing interests.

## Figures and Tables

**figs1:**
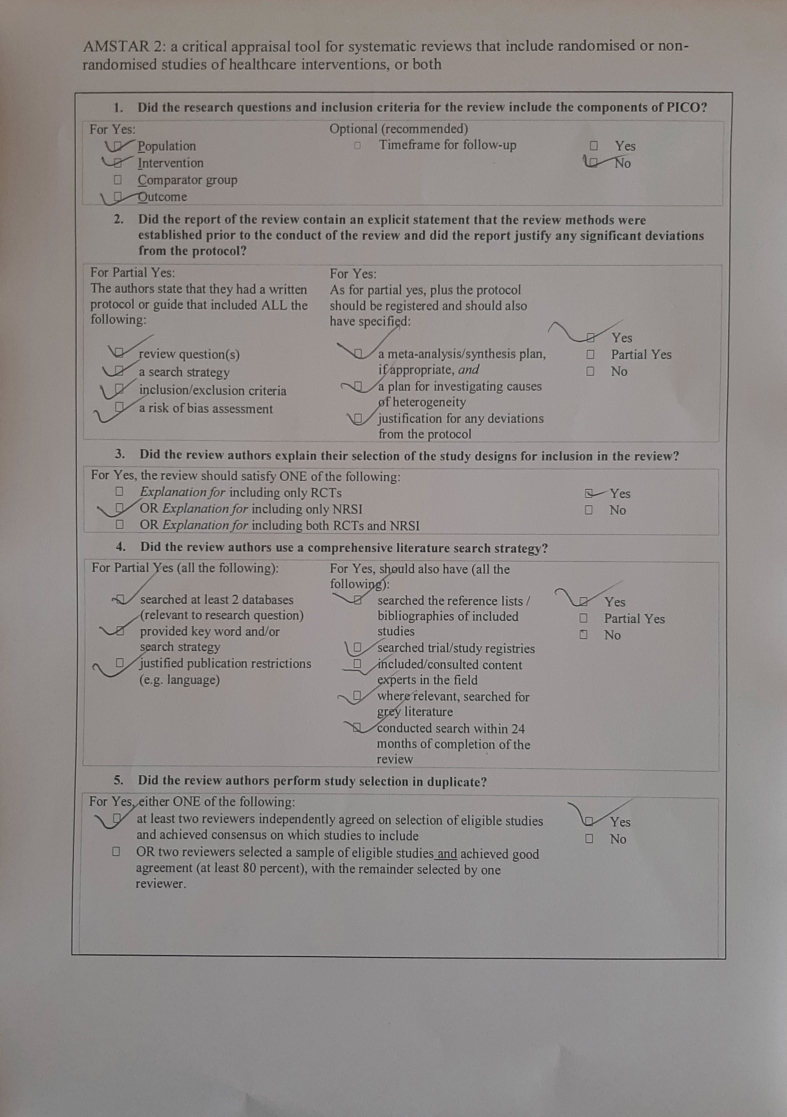


**figs2:**
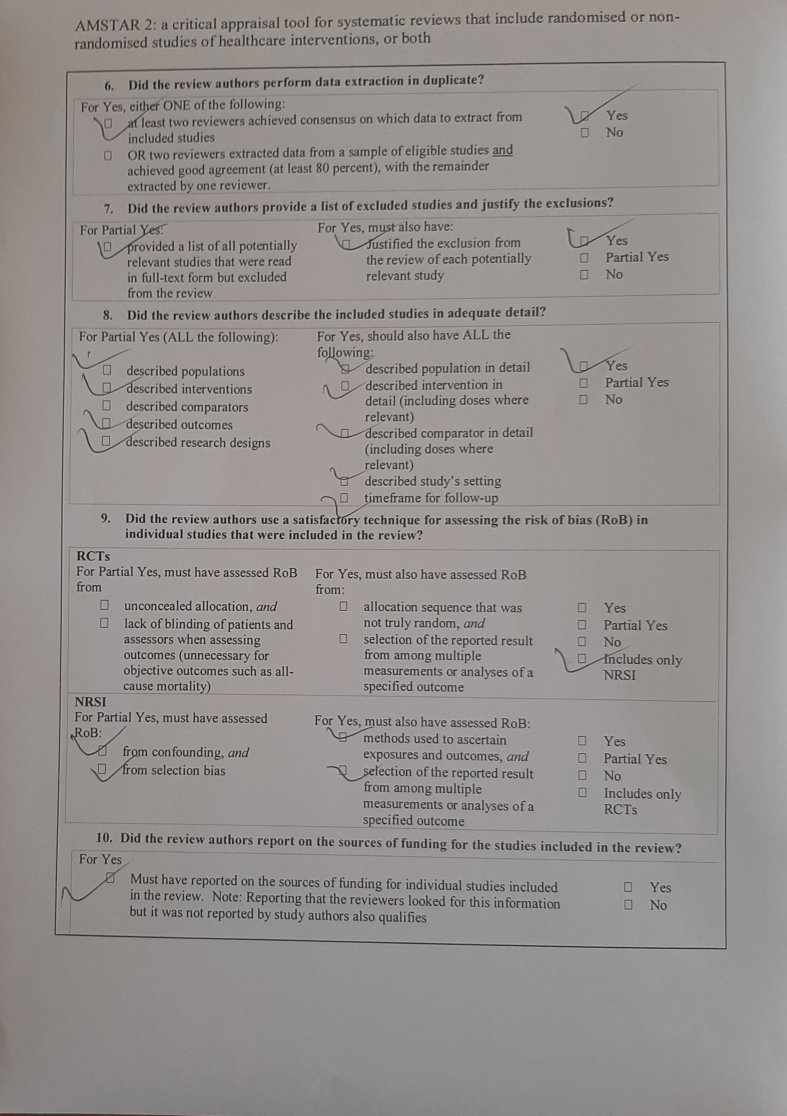


**figs3:**
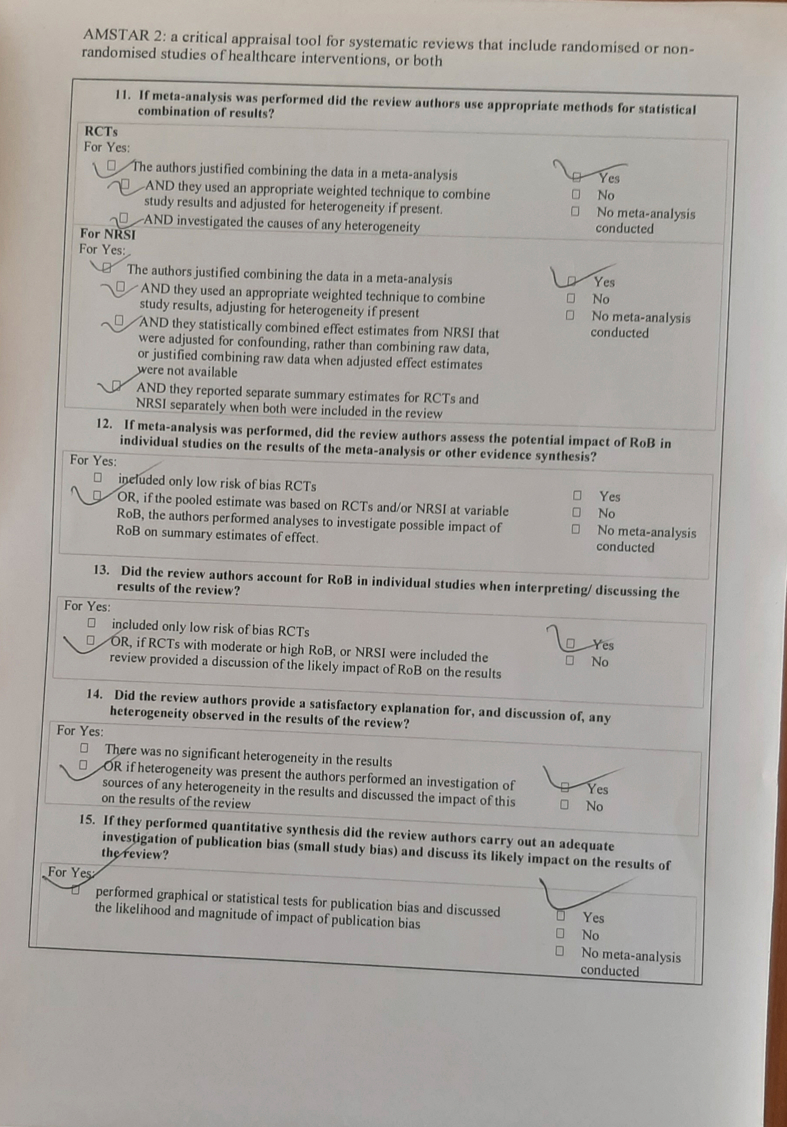


**figs4:**
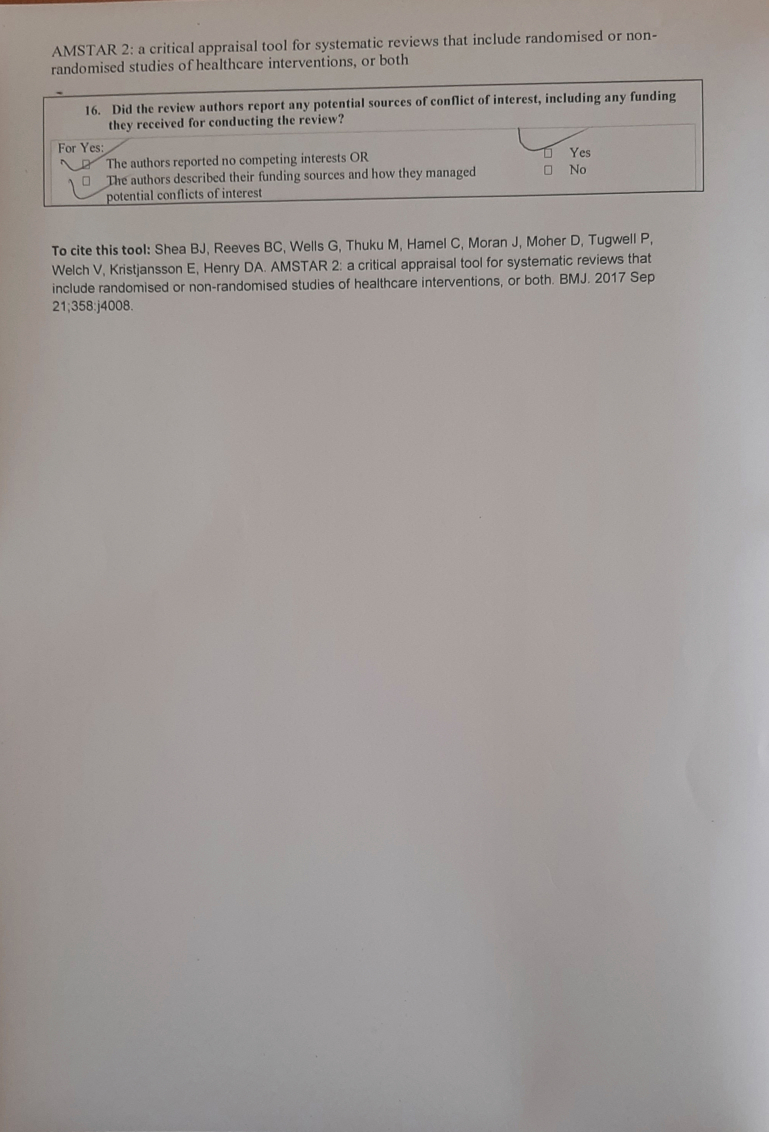


## Abbreviations

AMI: Acute Myocardial injury
BNP: Brain Nautric Peptide
CI: Confidence Interval
CINHAL: Cumulative Index to Nursing and Allied Health Literature
CK-MB: Creatinine Kinase Myocardial Band;
hs-TnT: Highly Sensitive Troponin
LILACS: Latin American and Caribbean Health Science Information Center
MOOSE: Meta-analysis of Observational Studies in Epidemiology
OR: Odds Ratio
PRISMA: Preferred Reporting Items for Systematic Reviews and Meta-Analyses
RR: Relative Risk
SD: Standard Deviation
