# Supplementary material for: Global burden of acute myocardial injury associated with COVID-19: A systematic review, meta-analysis, and meta-regression
Source: Ann Med Surg (Lond). 2021 Jul 28;68:102594. doi: 10.1016/j.amsu.2021.102594 (PMC8316689; doi:10.1016/j.amsu.2021.102594)
Supplement: Multimedia component 1 [file mmc1.doc]

| **Section/topic** | **#** | **Checklist item** | **Reported on page #** |
| --- | --- | --- | --- |
| **TITLE** | | |  |
| Title | 1 | Global burden of acute myocardial injury associated with COVID-19: A systematic review, Meta-analysis, and Meta-regression | 1 |
| **ABSTRACT** | | |  |
| Structured summary | 2 | **Abstract**  Background: The body of evidence showed that there is a strong correlation between acute myocardial Injury and COVID-19 infection. However, the link between acute myocardial infection and COVID-19, the prevalence, reliability of diagnostic modalities, independent predictors, and clinical outcomes are still uncertain and a topic of debate. The current study was designed to determine the prevalence, determinants, and outcomes of acute myocardial injury based on a systematic review and meta-analysis the global published peer-reviewed works of literature.  Methods: A comprehensive search was conducted in PubMed/Medline; Science direct, CINHAL, and LILACS from December 2019 to May 2021. All observational studies reporting the prevalence of AMI were included while case reports and reviews were excluded. The data were extracted with two independent authors in a customized format. The methodological quality of included studies was evaluated using the Newcastle-Ottawa appraisal tool.  Results: A total of 397 articles were identified from different databases. Thirty-seven Articles with 21, 204 participants were included while seven studies were excluded. The meta-analysis revealed that the pooled prevalence of myocardial injury during the COVID-19 pandemic was 22.33% (95% CI: 17.86 to 26.81, 37).  Conclusion: Our meta-analysis showed that mortality among patients with an acute myocardial injury during COVID-19 was more than four times more likely as compared to those without AMI. This necessitates a mitigating strategy to prevent and manage before its clinical outcomes getting worse  Registration: This systematic review was registered in Prospero's international prospective register of systematic reviews (CRD42021257184).  Keywords: Myocardial injury; Mortality; prevalence | 2 |
| **INTRODUCTION** | | |  |
| Rationale | 3 | The body of evidence showed that there is a strong correlation between acute myocardial Injury and COVID-19 infection. However, the link between acute myocardial infection and COVID-19, the prevalence, reliability of diagnostic modalities, independent predictors, and clinical outcomes are still uncertain and a topic of debate. | 3 |
| Objectives | 4 | The current study was designed to determine the prevalence, determinants, and outcomes of acute myocardial injury based on a systematic review and meta-analysis the global published peer-reviewed works of literature. | 4 |
| **METHODS** | | |  |
| Protocol and registration | 5 | The systematic review and meta-analysis was conducted based on the Preferred Reporting Items for Systematic and meta-analysis (PRISMA) protocols, and the Meta-analysis Of Observational Studies in Epidemiology (MOOSE*)* checklist. This systematic review and meta-analysis was registered in Prospero's international prospective register of systematic reviews (CRD42021257184) on May 27, 2021. | 5 |
| Eligibility criteria | 6 | All observational studies reporting the prevalence of myocardial injury among hospitalized patients with COVID-19 were included while studies that didn’t report the prevalence of myocardial injury among hospitalized patients with COVID-19, articles that didn’t report full information for data extraction, articles with different outcomes of interest, and Systemic review study design were excluded | 5 |
| Information sources | 7 | The search strategy was conducted to explore all available published and unpublished studies reporting myocardial injury among COVID-19 patients admitted to the hospital from December 2019 to May 2021 without language restrictions. A comprehensive search was employed in this review in different databases. An initial search on PubMed/Medline, Science Direct, CINHAL, and Cochrane Library was carried out followed by an analysis of the text words contained in Title/Abstract and indexed terms. A second search was undertaken by combining free text words and indexed terms with Boolean operators. The third search was conducted with the reference lists of all identified reports and articles for additional studies. | 5 |
| Search | 8 | COVID-19 OR novel coronavirus OR SARS-CoV-2 AND Myocardial injury OR myocardial damage OR myocardial infarction OR myocardial necrosis OR myocarditis OR myocardial dysfunction AND mortality OR death OR outcomes AND comorbidity OR complication AND prevalence OR incidence | 5 |
| Study selection | 9 | Studies were selected independently with two Authors with population, phenomena of interest, context and design | 6 |
| Data collection process | 10 | The data from each study were extracted by SA and YC independently with a customized format excel sheet. The disagreements between the two independent authors were resolved by the other authors. The extracted data included: Author names, country, date of publication, sample size, the prevalence of myocardial injury, mortality, the number of days in the hospital, presence of co-morbidities, mean and SD of cardiac biomarkers, and determinants | 6 |
| Data items | 11 | No special data items to be described and defined as it has been described in methodology. | 6 |
| Risk of bias in individual studies | 12 | Not applicable | 6 |
| Summary measures | 13 | The main summary measures was proportion, RR, and OR with 95% confidence interval | 6 |
| Synthesis of results | 14 | Data analysis was carried out in R statistical software version 4.0.2 and STATA 16. The pooled prevalence of myocardial injury, mortality, and length of hospital stay among hospitalized patients with COVID-19 was determined with a random effect model with restricted maximum likely hood (REML) as there was substantial heterogeneity. The Heterogeneity among the included studies was checked with forest plot, χ2 test, I^2^ test, and the p-values. Substantial heterogeneity among the included studies was investigated with subgroup analysis for categorical moderators (comorbidity, setting, country, and age group) and meta-regression for continuous covariates (mean troponin level, mean lengths of stay, mean age, and sample size) for outcomes extracted from more than ten studies. | 6 |

Page 1 of 2

| **Section/topic** | **#** | **Checklist item** | **Reported on page #** |
| --- | --- | --- | --- |
| Risk of bias across studies | 15 | We tried to assess publication bias with funnel plot with Egger’s test, beggs and trim fill | 6 |
| Additional analyses | 16 | Subgroup analysis and meta-regression was done to find out source of heterogeneity. Besides, factor analysis was done to adjust the potential confounders. | 6 |
| **RESULTS** | | |  |
| Study selection | 17 | A total of 397 articles were identified from different databases with an initial search. Forty-four articles were selected for evaluation after the successive screening. Thirty-seven Articles with 21, 204 participants were included in the systematic review and Meta-Analysis while thirteen studies were excluded with reasons | 7 |
| Study characteristics | 18 | Twenty-four of the included studies were conducted in China while three studies were conducted in the USA and four in Italy. One study was conducted at a multi-country level. The remaining studies were conducted in France, Denmark, and South Korea. The mean age (±SD) of the  Participants varied from 49.25±4.25 to 77±9 years. | 8 |
| Risk of bias within studies | 19 | Not applicable |  |
| Results of individual studies | 20 | The results of individual studies were narrated in result section | 8 |
| Synthesis of results | 21 | The synthesis of results was carried out with R software version 3.6.1. , and STATA 16 |  |
| Risk of bias across studies | 22 | Risk of bias was tried to be addressed with funnel plot |  |
| Additional analysis | 23 | Moderator and regression analysis were conducted |  |
| **DISCUSSION** | | |  |
| Summary of evidence | 24 | A total of 397 articles were identified from different databases. Thirty-seven Articles with 21, 204 participants were included while seven studies were excluded. The meta-analysis revealed that the pooled prevalence of myocardial injury during the COVID-19 pandemic was 22.33% (95% CI: 17.86 to 26.81, 37). . | 17 |
| Limitations | 25 | The meta-analysis included studies with were low-powered and the majority of included studies didn’t report data on mortality, comorbidity, and risk factors to investigate the independent predictors. Besides, the included studies used different cut points for the diagnosis of acute myocardial injury with cardiac biomarkers and it would be difficult to provide conclusive evidence | 18 |
| Conclusions | 26 | Our meta-analysis showed that mortality among patients with an acute myocardial injury during COVID-19 was more than four times more likely as compared to those without AMI. Besides, patients with a history of smoking, acute coronary disease, chronic obstructive pulmonary disease, and hypertension were independent predictors of acute myocardial injury. | 19 |
| **FUNDING** | | |  |
| Funding | 27 | Authors own resources | 20 |

*From:*  Moher D, Liberati A, Tetzlaff J, Altman DG, and the PRISMA Group (2009). Preferred Reporting Items for Systematic Reviews and Meta-Analyses: The PRISMA Statement. PLoS Med 6(7): e1000097. doi:10.1371/journal.pmed1000097

Page 2 of 2
