# Supplementary material for: Global burden of acute myocardial injury associated with COVID-19: A systematic review, meta-analysis, and meta-regression
Source: Ann Med Surg (Lond). 2021 Jul 28;68:102594. doi: 10.1016/j.amsu.2021.102594 (PMC8316689; doi:10.1016/j.amsu.2021.102594)
Supplement: Multimedia component 2 [file mmc2.docx]

**Supplemental Table 1: Methodological quality of included studies**

| Study | Selection | | | | Comparability | Outcome | | Quality Score |
| --- | --- | --- | --- | --- | --- | --- | --- | --- |
|  | Representativeness of the sample | Sample size calculation | Non-respondents | Ascertainment of confounders | Confounding factor controlled | Assessment of outcomes | Statistical tests |  |
| Cao et al, 2020 | 1 | 1 | 1 | 2 | 0 | 2 | 1 | 8 |
| Chen et al, 2020 | 1 | 1 | 1 | 2 | 0 | 2 | 1 | 8 |
| Deng et al, 2020 | 1 | 1 | 1 | 2 | 0 | 2 | 1 | 8 |
| Feng et al, 2020 | 1 | 1 | 1 | 2 | 0 | 2 | 1 | 8 |
| Ferrante et al, 2020 | 1 | 1 | 1 | 2 | 2 | 2 | 1 | 10 |
| Giustino et al, 2020 | 1 | 1 | 1 | 2 | 2 | 2 | 1 | 10 |
| Gramegna et al, 2020 | 1 | 1 | 1 | 1 | 0 | 2 | 1 | 7 |
| Han et al, 2020 | 1 | 1 | 1 | 0 | 1 | 1 | 1 | 7 |
| Haung et al, 2020 | 1 | 1 | 1 | 2 | 0 | 2 | 1 | 8 |
| Lala et al, 2020 | 1 | 1 | 1 | 2 | 2 | 2 | 1 | 10 |
| Li D et al, 2020 | 1 | 1 | 1 | 1 | 2 | 2 | 1 | 8 |
| Li et al, 2020 | 1 | 1 | 1 | 2 | 2 | 2 | 1 | 10 |
| Metkus et al, 2020 | 1 | 1 | 1 | 2 | 2 | 2 | 1 | 10 |
| Modin et al, 2020 | 1 | 0 | 1 | 1 | 2 | 1 | 1 | 7 |
| Popovic et al, 2020 | 1 | 1 | 1 | 1 | 0 | 2 | 1 | 7 |
| Richardson et al, 2020 | 1 | 1 | 1 | 2 | 0 | 2 | 1 | 8 |
| Shi et al, 2020 | 1 | 1 | 1 | 2 | 2 | 2 | 1 | 10 |
| Shi Q et al, 2020 | 1 | 1 | 1 | 2 | 0 | 2 | 1 | 8 |
| Shi S et al, 2020 | 1 | 1 | 1 | 2 | 2 | 2 | 1 | 10 |
| Stefanini et al, 2020 | 1 | 1 | 1 | 2 | 2 | 2 | 1 | 10 |
| Tu et al, 2020 | 1 | 1 | 1 | 1 | 0 | 1 | 1 | 6 |
| Wang et al, 2020 | 1 | 1 | 1 | 2 | 0 | 2 | 1 | 8 |
| Wang Y et al, 2020 | 1 | 1 | 1 | 1 | 0 | 2 | 1 | 7 |
| Wei et al, 2020 | 1 | 1 | 1 | 2 | 2 | 2 | 1 | 10 |
| Wu et al, 2020 | 1 | 1 | 1 | 2 | 2 | 2 | 1 | 10 |
| Xiong et al, 2020 | 1 | 1 | 1 | 2 | 0 | 2 | 1 | 8 |
| Yang et al, 2020 | 1 | 1 | 1 | 2 | 0 | 2 | 1 | 8 |
| Yuan et al, 2020 | 1 | 1 | 1 | 1 | 0 | 2 | 1 | 7 |
| Zhang et al, 2020 | 1 | 1 | 1 | 2 | 2 | 2 | 1 | 10 |
| Zhou et al, 2020 | 1 | 1 | 1 | 2 | 2 | 2 | 1 | 10 |
| Aggarwal et al, 2020 | 1 | 1 | 1 | 2 | 1 | 1 | 1 | 8 |
| Saleh et al, 2020 | 1 | 1 | 1 | 2 | 2 | 2 | 1 | 10 |
| Hong et al, 2020 | 1 | 1 | 1 | 2 | 0 | 2 | 1 | 8 |
| Javanian et al, 2020 | 1 | 1 | 1 | 2 | 1 | 2 | 1 | 9 |
| Lombardi et al, 2020 | 1 | 1 | 1 | 2 | 1 | 2 | 1 | 9 |
| Du et al, 2020 | 1 | 1 | 1 | 2 | 2 | 2 | 1 | 10 |
| Xu et al, 2020 | 1 | 1 | 1 | 2 | 2 | 2 | 1 | 10 |
